# Novel Pharmacological Approaches for Inflammatory Bowel Disease: Targeting Key Intracellular Pathways and the IL-23/IL-17 Axis 

**DOI:** 10.1155/2012/389404

**Published:** 2012-03-15

**Authors:** Leo R. Fitzpatrick

**Affiliations:** Department of Pharmacology, Penn State College of Medicine, Hummelstown, PA 17036, USA

## Abstract

This review identifies possible pharmacological targets for inflammatory bowel disease (IBD) within the IL-23/IL-17 axis. Specifically, there are several targets within the IL-23/IL-17 pathways for potential pharmacological intervention with antibodies or small molecule inhibitors. These targets include TL1A (tumor necrosis factor-like molecule), DR3 (death receptor 3), IL-23, IL-17 and the receptors for IL-23 and IL-17. As related to IBD, there are also other novel pharmacological targets. These targets include inhibiting specific immunoproteasome subunits, blocking a key enzyme in sphingolipid metabolism (sphingosine kinase), and modulating NF-*κ*B/STAT3 interactions. Several good approaches exist for pharmacological inhibition of key components in the IL-23 and IL-17 pathways. These approaches include specific monoclonal antibodies to TL1A, IL-17 receptor, Fc fusion proteins, specific antibodies to IL-17F, and small molecule inhibitors of IL-17 like Vidofludimus. Also, other potential approaches for targeted drug development in IBD include specific chemical inhibitors of SK, specific small molecule inhibitors directed against catalytic subunits of the immunoproteasome, and dual inhibitors of the STAT3 and NF-*κ*B signal transduction systems. In the future, well-designed preclinical studies are still needed to determine which of these pharmacological approaches will provide drugs with the best efficacy and safety profiles for entrance into clinical trials.

## 1. Introduction

During the past decade, there has been an expansion in new scientific knowledge related to the pathogenesis of inflammatory bowel disease (IBD). This knowledge has been summarized rather recently in published reviews, which provided key insights into IBD pathogenesis [1, 2]. Briefly, IBD consists of two distinct diseases, Crohn's disease (CD) and ulcerative colitis (UC). CD and UC are thought to arise due to a combination of genetic variations and alterations in the bacterial flora, which can subsequently drive a dysregulated immune response that results in chronic intestinal inflammation [1, 2]. 

Recent information related to the pathogenesis of IBD has provided the rationale for new pharmacological approaches to better treat the intestinal inflammation and related symptoms in patients. Another scientific review has succinctly summarized current therapies for IBD: mesalazine-based drugs, corticosteroids, immunosuppressive drugs (azathioprine/6-mercaptopurine, methotrexate, cyclosporin, anti-TNF agents), as well as emerging biologic agents such as antiadhesion and antiintegrin molecules [3]. 

This review will primarily focus on possible pharmacological targets within the IL-23/IL-17 proinflammatory pathway (i.e., IL-23/IL-17 Axis), including some work from our laboratory [4]. Secondarily, this review will provide insights into some other novel pharmacological targets, such as inhibiting specific immunoproteasome subunits, blocking a key enzyme in sphingolipid metabolism (sphingosine kinase), and modulating NF-B/*κ*STAT3 interactions. Scientific data supporting these pharmacological targets will be provided from the published literature [5–12].

**Figure 1:**
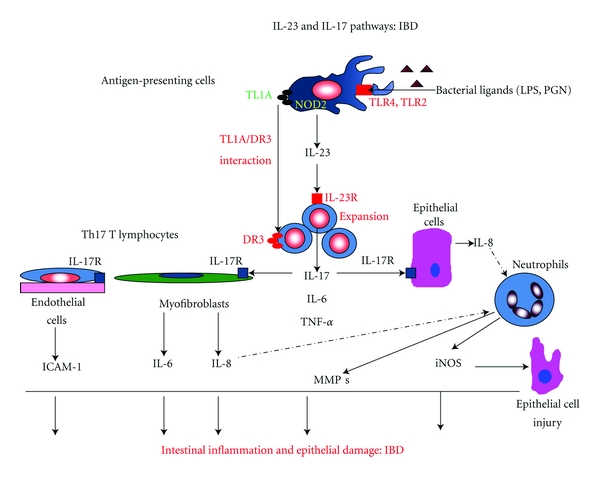
This figure shows relevant cell types, mediators, and potential pharmacological targets associated with IL-23 and IL-17 pathways (IL-23/IL-17 Axis), which are operative within the context of inflammatory bowel disease (IBD). Bacterial ligands (lipopolysaccharide [LPS] and peptidoglycan [PGN]) bind to their respective toll-like receptors (TLR4 and TLR2) and induce IL-23 release from antigen-presenting cells (APC's). IL-23 binds to the IL-23 receptor (IL-23R) to stimulate expansion of Th-17-producing cells, which release IL-17. In addition, interactions between TL1A (tumor necrosis factor-like molecule) on APC's and DR3 (death receptor 3) on T lymphocytes induces the secretion of IL-17. These pathways also promote the secretion other proinflammatory cytokines like IL-6 and TNF-*α*. IL-17 stimulates the expression of adhesion molecules (e.g., ICAM-1) on endothelial cells, as well as the release of IL-6 and IL-8 from myofibroblasts and epithelial cells. IL-8 acts as a chemotactic factor for neutrophil influx into the intestine. Infiltrating neutrophils release inflammatory mediators like matrix metalloproteinases (MMP's) and inducible nitric oxide synthase (iNOS). This sequelae of pathogenic events leads to the chronic inflammation and epithelial cell damage associated with IBD.

There are several targets within the IL-23/IL-17 pathways for potential pharmacological intervention with antibodies or small molecule inhibitors. These targets include TL1A, DR3, IL-23, IL-23R, IL-17, and IL-17R (Figure 1). 

## 2. TL1A/DR3

As shown in Figure 1, upstream binding of bacterial derived ligands such as lipopolysaccharide (LPS) and peptidoglycan (PGN) to their specific toll-like receptors (TLR4 and TLR2, respectively) can induce TL1A (tumor necrosis factor-like molecule) expression in antigen presenting cells (like dendritic cells) [13]. Downstream, the interaction of TL1A with DR3 (death receptor 3) results in the production of IL-17 from Th17 T lymphocytes [14].

The interaction between this TNF-family member (TL1A) and its receptor DR3 plays an important role in autoimmune diseases such as experimental autoimmune encephalomyelitis (EAE) [15]. More recently, other investigators have published an informative review on the role of the TL1A-DR3 pathway in the pathogenesis of IBD [14]. Of note, TL1A expression is increased in the inflamed intestinal tissue of patients with CD [1]. 

In 2008, Takedatsu and colleagues showed that TL1A and DR3 expression was upregulated in the gut-associated lymphoid tissue (GALT) of mice with chronic dextran sulfate sodium (DSS)-induced colitis [16]. Importantly, from a pharmacological standpoint, a monoclonal antibody (mAb) to TL1A effectively attenuated chronic DSS-induced colitis, as well as T-cell transfer colitis in mice [16]. This antibody also improved established chronic colitis. The anticolitis effects were associated with decreases in IFN-*γ*, IL-17, and IL-6 production from GALT [16]. These results clearly established targeting of TL1A, as a rational pharmacological approach for IBD. More recently, two other research groups have generated transgenic mice with enhanced expression of TL1A in T-cells or dendritic cells [17, 18]. These mice developed predominantly small intestinal pathology, which was dependent upon DR3, IL-13, and IL-17 [17, 18]. Important studies were then carried out in mice with acute trinitrobenzenesulfonic acid (TNBS)-induced colitis. These mice were treated with an antagonistic mAb to TL1A, a DR3-Fc fusion protein, or an antagonistic mAb to DR3 [17]. Mice treated with anti-TL1A showed a marked improvement in indices of TNBS-induced colitis. Also, partial protection against this murine colitis was found with the anti-DR3 pharmacological approaches [17]. Taken as a whole, these results further suggest that targeting of the TL1A-DR3 pathway could be a good pharmacological approach for both types of human IBD (CD and UC) [17–19].

## 3. IL-23

As shown in Figure 1, upon stimulation by appropriate ligands, IL-23 is produced by antigen-presenting cells. After binding to the appropriate receptor (IL-23R), this cytokine can stimulate the production of IL-17, TNF-*α*, and IL-6 from T-cells. Therefore, IL-23 was proposed to play an integral role in the pathogenesis of IBD [20]. From a potential therapeutic standpoint, Elson and colleagues created T-cell transfer colitis in SCID mice recipients with bacterial reactive Th17 CD4+ T-cells [21]. Treatment of these mice with an antibody to the p19 subunit of IL-23 both prevented T-cell transfer colitis and effectively treated established colitis [21]. This is a rather specific therapeutic approach for treating IBD, because only the p19 subunit is targeted. This subunit is endogenous only to IL-23 but is not shared by IL-12, like the common p40 subunit [21]. An antibody targeting the common p40 subunit (Ustekinumab) has shown some evidence of efficacy in patients with CD (phase II a trial) and is undergoing further clinical trials [22, 23]. Ustekinumab was generally well tolerated in these IBD patients [22, 23]. In the long term, it remains to be determined whether an antibody targeting solely IL-23 p19 will have a better efficacy/safety ratio than Ustekinumab in IBD patients [21–23]. 

In order to investigate a downstream component of the IL-23 pathway, Takedatsu et al. determined whether a mAb to the IL-23 receptor (IL-23R) attenuated indices of acute or chronic-DSS-induced colitis in mice [16]. Interestingly, the chronic phase of colitis was attenuated by treatment with the IL-23 mAb to a greater degree than the acute phase of colonic inflammation [16]. Furthermore, the anticolitis effects with the IL-23 mAb seemed to be less dramatic than the effects with the mAb to TLA1 [16]. As suggested by the authors, it is possible that neutralizing TL1A could induce more comprehensive effects than just blocking downstream components of the IL-23/IL-17 axis (Figure 1) [14–16]. Therefore, in addition to affecting IL-17 production (Figure 1), blocking the IL-12/IFN-*γ* pathway by TL1A neutralization may also be needed to effectively treat the colonic inflammation associated with human IBD [16]. Interestingly, it has recently been reported that, in CD patients, there is a population of CD 161^(+)^ CD4 T-cells which produce both IL-17 and IFN-*γ* [24]. As a whole, these results emphasize the complexity in the pathogenesis of IBD, involving multiple inflammatory mediators. This complexity must be recognized within the context of developing novel pharmacological approaches for UC and CD. 

## 4. IL-17

Elevated expression of IL-17 has been reported in the inflamed intestine of patients with UC and CD [2, 24]. IL-17, which is the prototypical cytokine produced by Th17 cells, plays a potential role in the amplification of intestinal inflammation. Specifically, IL-17 stimulates various cell types (endothelial cells, myofibroblasts, and epithelial cells) to produce proinflammatory mediators that amplify intestinal inflammation (Figure 1) [25, 26]. Therefore, it is interesting that variable and somewhat contrasting results have been obtained with approaches that inhibit the function of IL-17 in animal models of IBD [25–28]. These contrasting results could be related to different functions of IL-17A and IL-17F, within the specific context of intestinal inflammation [25–27]. In this regard, Yang and colleagues showed that murine DSS-induced colitis was worsened in IL-17A knockout (KO) mice but significantly improved in IL-17F KO mice [27]. Furthermore, a protective role was also proposed for IL-17A in a T-cell transfer model of colitis [26, 28]. In contrast, Zhang and colleagues showed that acute TNBS-induced colitis was attenuated in IL-17 receptor (IL-17R) KO mice, as well as in animals treated with an IL-17 R:Fc fusion protein [25]. It is probable that the IL-17 R KO mice would not respond to either IL-17A or IL-17F, suggesting that inhibition of both forms of IL-17 is needed for attenuation of colitis [25]. 

Vidofludimus (4SC-101) is a novel small molecule inhibitor of dihydroorotate dehydrogenase (DHODH), which is a key enzyme involved in pyrimidine (i.e., uridine biosynthesis) in activated lymphocytes [4]. However, our research group showed that Vidofludimus inhibited IL-17 production in activated lymphocytes, even in the presence of exogenous uridine. Our results suggested a pharmacological effect that was independent of inhibiting DHODH and T-cell proliferation [4]. Subsequently, we showed that Vidofludimus could inhibit IL-17 secretion in activated splenocytes by inhibiting STAT3 and NF-*κ*B-signaling pathways [29]. Importantly, Vidofludimus attenuated various parameters of acute TNBS-induced colitis in mice, including IL-17 production [4]. Specifically, this anticolitis profile was associated with a reduction in the colonic expression of both IL-17 A/A homodimers, as well as IL-17 F/A heterodimers [4]. These results suggested that Vidofludimus would be an appropriate drug for use in patients with IBD. Indeed, in a recent Phase II European clinical trial, Vidofludimus demonstrated a good efficacy and safety profile in patients with IBD [30]. Because this small molecule compound has the potential for inhibiting T-lymphocyte proliferation, as well as inhibiting relevant IL-17A and IL-17F signal transduction pathways, it is an interesting candidate for future clinical studies. 

Finally, with regard to IL-17 inhibition, AIN457 (Secukinumab) is a human anti-IL-17A antibody that has been developed by Novartis Healthcare [26]. Based on an oral presentation at the 2011 Digestive Disease Week meeting, it seems that recent clinical results in CD patients treated with AIN457 have been negative. Specifically, Secukinumab-treated patients did not show improvement in parameters of disease [31]. At first glance, these results seem to be counterintuitive to the schematic pathways in Figure 1. However, plasma levels of IL-17F, as well as IL-17F production by stimulated splenocytes, are elevated in IL-17A-deficient mice [32]. In this regard, the preclinical literature suggests that specifically inhibiting IL-17F, and/or inhibiting both IL-17 A and IL-17F, may be necessary to achieve good anticolitis actions [4, 25, 27].

## 5. Novel Intracellular Signaling Targets for IBD

**Figure 2:**
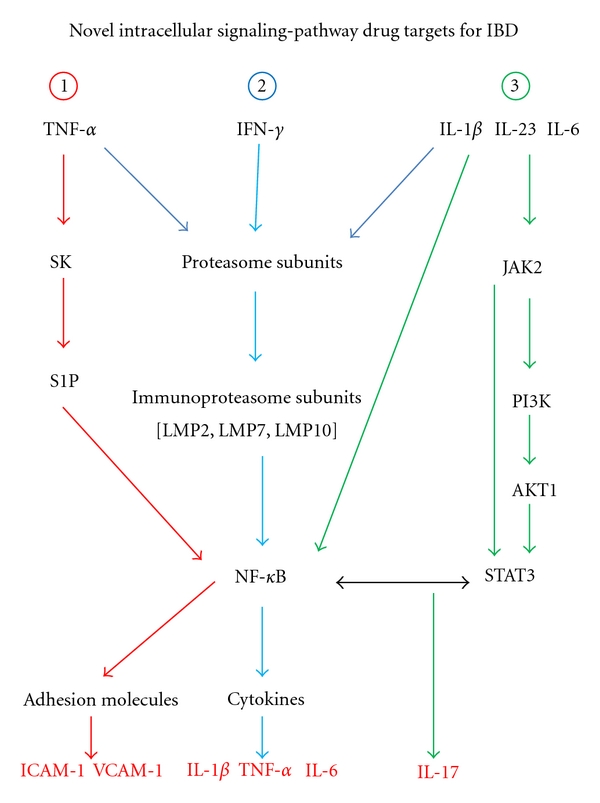
This figure shows three novel intracellular signaling pathways involved in the pathogenesis of IBD. Pathway 1: TNF-*α* induces adhesion molecule expression in endothelial cells, as well as proinflammatory cytokine (IL-1*β*, IL-6) production by monocytes, through a sphingosine kinase (SK), sphingosine-1-phosphate (S1P), nuclear factor-kappa B (NF-*κ*B)-dependent pathway. Pathway 2: upon stimulation of cells with proinflammatory cytokines (IFN-*γ*, TNF-*α*, and IL-1*β*), constitutive proteasome subunits are converted to the immunoproteasome subunits *β*1i (LMP2), *β*2i (LMP10, MECL-1), and *β*5i (LMP7) [38–40]. Functionally, immunoproteasome subunits play a role in NF-*κ*B signaling. Pathway 3: dual activation of NF-*κ*B and STAT3 pathways controls the expression of IL-17. As shown in this figure, crosstalk between these three pathways occurs, thereby promoting intestinal inflammation. Specific components of these pathways such as sphingosine kinase (SK), immunoproteasome subunits (LMP2, LMP7, and LMP10), and interactions between NF-*κ*B/STAT3 represent possible pharmacological targets for IBD. In the figure: LMP is low molecular mass polypeptide (2, 5, or 10); JAK2 is Janus Kinase 2; PI3K is phosphoinositide-3 kinase; AKT1 is Alpha serine/threonine-protein kinase.

Figure 2 shows three intracellular signaling pathways that are potentially involved in the pathogenesis of IBD: (1) altered sphingolipid metabolism, whereby the enzyme sphingosine kinase (SK) appears to play a critical role in signaling by TNF-*α* [9–11], (2) upregulation of immunoproteasome subunits by proinflammatory cytokines, which downstream is connected to activation of the NF-*κ*B signal transduction system [5–8, 33–35], and (3) dual activation of NF-*κ*B and STAT3 signal pathways by cytokines, which results in enhanced IL-17 production by leukocytes [29, 36, 37]. These pathways are summarized in Figure 2. Interestingly, as shown in this figure, crosstalk between inflammatory pathways occurs, which likely promotes intestinal inflammation.

Based on the pathways outlined in Figure 2, this section of the review will specifically focus on three pharmacological targets for IBD: (1) inhibition of SK, (2) inhibiting specific catalytic subunits of the immunoproteasome, and (3) modulating NF-*κ*B/STAT3 interactions. 

## 6. SK Inhibition

SK is involved in the conversion of sphingosine to sphingosine-1-phosphate (S1P) [9–11]. Importantly, SK exists as two isoforms (SK1 and SK2), with diverse biological functions, which have been reviewed elsewhere [41, 42]. A critical step in the mechanism of action for TNF-*α* includes the activation of SK [9–11, 41, 42]. Of critical relevance to this review, SK signals downstream through activation of the transcription factor NF-*κ*B (Figure 2, pathway 1). Specifically, *in vitro* studies have shown that TNF-*α* induces adhesion molecule expression in endothelial cells, as well as proinflammatory cytokine (IL-1*β*, IL-6) production by monocytes, through an SK-SIP-NF-*κ*B-dependent pathway (Figure 2) [9, 43, 44]. Recent results have shown that SK1 expression was increased in colonic tissue samples from patients with UC [11]. The potential role that SK plays in the generation of proinflammatory molecules relevant to the pathogenesis of IBD has prompted investigators to evaluate whether SK inhibition can effectively attenuate intestinal inflammation. 

Snider et al. showed that DSS-induced colitis was less severe in SK-1-deficient (SK1^−/−^) mice compared to wild-type control mice [11]. From a pharmacodynamic standpoint, intestinal SK1 mRNA expression, as well as SK activity (generation of S1P) were both attenuated in SK1-deficient mice. These results suggest that specific inhibition of SK1 may represent a valid pharmacological approach for IBD [11]. Maines and colleagues showed that treatment of mice with ABC249640 (a selective small molecule inhibitor of SK2) effectively attenuated parameters of murine DSS-induced colitis, as well as TNBS-induced colitis in mice and rats [9, 10]. Treatment of mice with ABC294640 resulted in reduced colonic S1P levels, as well as decreased levels of proinflammatory cytokines (IL-1*β*, IL-6, TNF-*α*, and IFN-*γ*) [9, 10]. Interestingly, these investigators found that this small molecule inhibitor also potently inhibited TNF-*α*-induced NF-*κ*B activation *in vitro* [9]. As a whole, these results suggest that inhibiting SK2 may also represent a good therapeutic approach for IBD [9, 10]. Since SK1 and SK2 are reported to have different biological actions on cellular proliferation and apoptosis [42, 43], it remains to be determined as to which SK isoform represents the best pharmacological target for IBD [9–11]. Nevertheless, targeting the SK pathway (Figure 2) seems to be a rational therapeutic approach for IBD. 

## 7. Inhibition of Immunoproteasome Subunits

The constitutive 20S proteasome has a cylindrical structure consisting of three catalytic subunits (*β*1, *β*2, and *β*5). Upon stimulation of cells with proinflammatory cytokines (IFN-*γ* and TNF-*α*), these constitutive subunits are converted to the immunoproteasome subunits *β*1i (LMP2), *β*2i (LMP10 or MECL-1), and *β*5i (LMP7) [38–40]. Functionally, immunoproteasome subunits play a role in MHC class I antigen presentation, as well as NF-*κ*B signaling [40, 45–47].

Over the past five years, several research groups (including our own) have suggested a potential role for the immunoproteasome subunits in the pathogenesis of both murine colitis and human IBD [5–8, 33–35]. We showed enhanced expression of the LMP2 (low molecular mass polypeptide 2) subunit in patients with active IBD, particularly in CD patients. Interestingly, LMP2 was also upregulated in areas of the intestine devoid of macroscopic disease [5]. Generally, our results were confirmed by other investigators, who showed significantly enhanced levels of LMP2, LMP7, and LMP10 in CD patients [33–35, 48]. Importantly, in patients with CD, upregulation of the NF-*κ*B signal transduction system was observed in the inflamed intestinal mucosa [33].

Using LMP2 knockout mice, we showed that various parameters of DSS-induced colitis (including colonic IL-1*β*) were improved compared to WT control mice [6]. Schmidt and colleagues found that parameters of DSS-induced colitis were also attenuated in LMP7-deficient-mice [8]. In these mice, there was diminished activation of the NF-*κ*B signal transduction system, resulting in less expansion of Th1 and Th17 T-cells [8]. Basler et al. extended these findings. They showed that mice deficient in any of the immunoproteasome subunits (LMP2, LMP7, and MECL-1) had significant improvements in multiple indices of DSS-induced colitis [7]. Interestingly, significantly reduced levels of Th1 and Th17 cytokines were found in the LMP-deficient mice [7]. As a whole, these data suggest that targeting specific LMP subunits may represent a novel and effective pharmacological strategy for IBD (Figure 2, pathway 2). 

From a practical standpoint, targeting specific LMP subunits might best be done by novel chemical inhibitors. Importantly, it has already been shown that treatment with a selective inhibitor of LMP7 (PR-957) strongly suppressed murine DSS-induced colitis [8]. A drug development strategy, using specific LMP proteasome inhibitors (like PR-957), may provide good efficacy in IBD without the side effects of nonselective inhibitors like bortezomib, which also inhibits the constitutive subunits of the proteasome [5–8]. A specific chemical inhibitor of LMP2, designated as UK-101, has also been developed by a research group at the University of Kentucky [49]. This compound should also be tested in animal models of IBD. Finally, selective immunoproteasome inhibitors need to be tested in other colitis models, beyond the testing that has already been completed in the DSS model [8]. Results from these preclinical studies should allow the identification of optimal compound(s) to be progressed into clinical trials for IBD.

## 8. Inhibition of NF-*κ*B/STAT3-Signaling Pathways

It has been well documented in the literature that the NF-*κ*B pathway, as well as the STAT3 pathway, could be critically involved in the pathogenesis of IBD. Importantly, these papers delineate the roles of these pathways in mediating intestinal inflammation. This literature also points out potential drawbacks of inhibiting NF-*κ*B in epithelial cells, as well as blocking STAT3 in epithelial cells and innate immune cells [50–56].

Recently, intriguing information has also been published regarding dual activation of NF-*κβ* and STAT3 pathways in pathological conditions such as hepatic inflammation and cancer [12, 57, 58]. It is evident from Figure 2 that NF-*κ*B and STAT3 dually control the expression of some target genes (e.g., IL-17), thereby facilitating inflammation [12, 55, 57, 58]. Specifically, it was shown that the canonical NF-*κ*B pathway (involving I*κ*B-*α* degradation) and the STAT3 pathway (involving JAK2, PI3K, and AKT1 activation) are both activated by splenic-derived T-cell populations, following dual stimulation with IL-1*β* plus IL-23 (Figure 2) [36, 37]. Sutton and colleagues demonstrated that STAT3 and NF-*κ*B pathways mediated IL-17 production from *γδ* T-cells [37, 59]. Subsequently, these investigators reported that both *γδ* and CD4+ T-cells (via IL-17 production) promoted experimental autoimmune encephalomyelitis (EAE) in mice [59].

It is probable that interactions between the NF-*κ*B and STAT3 pathways could also contribute to the pathogenesis of intestinal inflammation/IBD (Figure 2, pathway 3) [12]. Indeed, activation of these pathways was described in conjunction with DSS-induced colitis in mice, as well as in murine TNBS-induced colitis [29, 60]. From a pharmacological development standpoint, there are two key questions that remain to be answered. (1) Are there any small molecule inhibitors that would be good candidates to inhibit interactions between NF-*κ*B and STAT3? (2) Would inhibition of these pathways be beneficial? 

Indeed, Youn et al. showed that treatment of mice with two plant-derived polyphenols (resveratrol and piceatannol) resulted in the attenuation of DSS-induced colonic inflammation, as well as downregulation of activated NF-*κ*B and STAT3 [60]. More recently, we have found that treatment with Vidofludimus attenuated the activation of STAT3 and NF-*κ*B pathways, as well as IL-17 production, in murine splenocytes and TNBS-induced colitis [5, 29]. The anticolitis effects that were observed with these chemical compounds are encouraging. However, resveratrol and piceatannol have antioxidant properties, while Vidofludimus can inhibit T-cell proliferation [5, 29, 60]. Therefore, further preclinical colitis studies need to be performed with more specific dual inhibitors of NF-*κ*B and STAT3, in order to gauge the clinical potential of this pharmacological approach for IBD. In this regard, a triterpenoid C28 methyl ester derivative (CDDO methyl ester) is an inhibitor of STAT3 (by preventing STAT3 phosphorylation), as well as an inhibitor of NF-*κ*B (by inhibiting I*κ*B kinase and downstream components of this signal transduction pathway) [61, 62]. Moreover, triterpenoids were effective in preclinical models of pancreatic cancer and cystic fibrosis lung disease [63, 64]. Therefore, CDDO methyl ester, or similar compounds, would be good candidates for testing in preclinical models of IBD.

In summary, all of the potential pharmacological targets (Figures 1 and 2) discussed in this review are upregulated in patients with IBD. Therefore, based on the preponderance of current data, several good opportunities exist for pharmacological inhibition of key components in the IL-23 and IL-17 pathways (Figure 1). These approaches include (1) specific mAb's to TL1A, (2) IL-17 R:Fc fusion proteins, (3) specific antibodies to IL-17F, and (4) small molecule inhibitors like Vidofludimus. Also, other potential opportunities for targeted drug development in IBD include specific chemical inhibitors of SK, specific small molecule inhibitors directed against catalytic subunits of the immunoproteasome, and dual inhibitors of the STAT3 and NF-*κ*B signal transduction systems (Figure 2). 

In the near future, critically designed preclinical studies are still needed to determine which of these pharmacological approaches will provide drugs with the best efficacy and safety profiles for entrance into clinical trials. Subsequently, well-designed clinical trials are needed to determine the specific pharmacological approaches that will prove to be most successful in patients with IBD.
